# Pruritogens in pemphigoid diseases: Possible therapeutic targets for a burdensome symptom

**DOI:** 10.1111/1346-8138.16652

**Published:** 2022-12-07

**Authors:** Sho Hiroyasu, Jay‐V James G. Barit, Aoi Hiroyasu, Daisuke Tsuruta

**Affiliations:** ^1^ Department of Dermatology Osaka Metropolitan University Graduate School of Medicine Osaka Japan

**Keywords:** bullous pemphigoid, itch, pemphigoid diseases, pruritogens, pruritus

## Abstract

Pruritus is a hallmark feature in pemphigoid diseases, where it can be severe and greatly impact the quality of life of affected patients. Despite being a key symptom, the exact pathophysiological mechanisms involved in pruritus in pemphigoid are yet to be fully elucidated and effective therapies addressing them are limited. This review summarizes the present understanding of pruritus specific to pemphigoid diseases, especially the pruritogens that induce it, and the therapeutic options that have been explored so far. The majority of the available evidence is on bullous pemphigoid and epidermolysis bullosa acquisita. Histamine derived from basophils correlates with pruritus severity, with omalizumab demonstrating promising efficacy in pruritus for bullous pemphigoid. IL‐4/−13 contribute to itch in bullous pemphigoid with dupilumab being evaluated in clinical trials. Other pruritogens of interest include substance P, tryptase, and thymic stromal lymphopoetin, with therapies targeting them requiring further investigation. Scratching behaviors contribute directly to blister formation through various mechanisms, such as pathological autoantibody recruitment, T helper cell type 1 polarization, and exposure of intracellular autoantigens. Treatments addressing these pathways may contribute to decreasing disease severity. Additional studies are needed to fully characterize how pruritus is regulated in pemphigoid diseases, to help pave the way to develop novel and effective therapeutics that will not only address pruritic symptoms but also decrease disease severity.

## 1 INTRODUCTION

Pruritus, also known as itch, is classically defined as an uncomfortable sensation that provokes a desire to scratch.^1^ Mild temporal pruritus is non‐pathological and physiologically functional to remove external elements from one's own body. On the other hand, severe chronic pruritus is pathological and significantly affects quality of life.^2^ Moreover, recurring pruritus leads to repeated scratching (itch–scratch cycle), aggravating disease morbidity by further damaging the skin and increasing proinflammatory cytokine release.^3^ Therefore, the treatment of pruritus is important not only for relief from the uncomfortable sensation itself, but also for stopping the itch–scratch cycle, preventing disease exacerbation.

Pemphigoid diseases (PDs) are a group of disorders characterized by tense blisters and edematous erythema, which include multiple diseases associated with severe pruritus, such as bullous pemphigoid (BP), epidermolysis bullosa acquisita (EBA), pemphigoid gestationis (PG), and linear IgA dermatosis (LAD).^4^ Treatment targeting pruritus in PDs is important since itch in PDs is often intractable and significantly impairs patients' quality of life (QOL), with scratch behaviors pathomechanistically contributing to blister formation.^5^, ^6^, ^7^ Pruritus treatment is also important in preclinical stages and several non‐bullous variants of PDs, wherein itch can be the sole manifestation.^8^ For these cases, the use of aggressive non‐specific immunosuppressive treatments such as systemic corticosteroids may be debatable due to its numerous deleterious adverse effects; and ideally, milder anti‐inflammatory or anti‐pruritic agents are preferred. In PDs, addressing pruritus both in preclinical and severe stages is clinically valuable, but the available therapeutic modalities are limited.

In other pruritic diseases, notably in atopic dermatitis, research advances elucidating the mechanisms of pruritus involving IL‐4, −13, and − 31 has resulted in the development of targeted therapies that revolutionized current clinical treatment.^9^, ^10^ While this drastic change is yet to be realized in the management of pruritus in PD, multiple pruritogens in PDs have been characterized in recent studies, which serve as potential therapeutic targets. This review summarizes the present understanding of the various mechanisms of pruritus in PDs, allowing a critical evaluation of possible more effective and novel therapeutic approaches addressing pruritus in these disorders.

## 2 CHARACTERISTICS AND PATHOLOGY OF PEMPHIGOID DISEASES

The PDs represent the subgroup of autoimmune blistering disorders that have autoantibodies against proteins at the dermal–epidermal junction (DEJ),^4^ comprised of BP, EBA, PG, LAD, mucous membrane pemphigoid (MMPh), and their other rare variants. Dermatitis herpetiformis is not included as its autoantigens do not localize at the DEJ and will not be discussed in this article.

While the autoantigen(s) vary for each PD, all PDs demonstrate autoantibody deposition at the DEJ.^4^ Most of these autoantigens are components or associated proteins of dermal–epidermal anchoring complexes termed hemidesmosomes. While the exact mechanisms leading to immune tolerance breakdown in PDs are yet to be fully elucidated, once autoantibodies are produced, they accumulate onto the DEJ, which is followed by complement recruitment and subsequent inflammatory infiltration.^11^ These inflammatory cells at the upper dermis secrete proteases such as neutrophil elastase, matrix metalloprotease‐9 (MMP‐9), plasmin, and granzyme B, which directly and indirectly induce DEJ protein cleavage, decreasing the attachment strength between the epidermis and dermis thus resulting in subepidermal blister formation.^12^, ^13^, ^14^, ^15^ Additionally in BP, binding of IgG type autoantibodies to the autoantigen, collagen XVII (COL17), induces its internalization into keratinocytes, contributing to reduced epidermal‐dermal adhesion.^16^, ^17^

## 3 CHARACTERISTICS OF PRURITUS IN PEMPHIGOID DISEASES

Recently, two independent studies analyzed the clinical characteristics of pruritus in newly diagnosed BP patients.^5^, ^18^ In both studies, daily pruritus is experienced by more than 75% of cases with a mean pruritus intensity of 5–6 out of 10. In one study, half of the participants had already been treated with antipruritic agents, suggesting that the current treatments strategies for addressing pruritus in PD (e.g. antihistamines) may be ineffective.^18^ Pruritus was mainly distributed at the upper arms, thighs, and back. Stress, sweating, and heat aggravate pruritus, while cold alleviates it. Pruritus was frequently associated with stinging and burning sensation, suggesting involvement of inflammatory mediators rather than neuropathic factors. Symptoms were more prominent in the evening and nighttime than in the morning and afternoon, with disturbed sleep being experienced by more than half of BP patients. Bullous pemphigoid disease activity index (BPDAI) scores, presence of eosinophilia, and serum anti‐COL17 or anti‐BP230 autoantibody titers did not correlate with pruritus severity, suggesting that pruritus in BP may have its own mechanism partially independent from blistering and these immunological factors.

Other pemphigoid diseases such as LAD, PG, inflammatory type of EBA (BP‐like EBA), and nonbullous pemphigoid are all recognized to have accompanying pruritus.^19^, ^20^, ^21^, ^22^ Probably because of their rarity, there are no distinct studies examining pruritus in these diseases thus far.

## 4 MECHANISMS OF PRURITUS

Generally, skin pruritus is triggered by pruritogens stimulating sensory nerve endings at the epidermis and upper dermis.^23^ The pruritus sensitive‐neurons are the unmyelinated C‐ and thinly myelinated Aδ‐fibers. These neuronal fibers convey itch signals via the dorsal root ganglion (DRG) and their central projections to the dorsal horn of the spinal cord. These signals are then transmitted via the thalamus to multiple brain regions, producing the itching sensation and eliciting scratch behavior.

Since PDs are skin‐specific inflammatory diseases with pruritus often localized particularly at the inflammatory skin lesions,^5^ most of the available pruritus studies in PDs investigated exclusively peripheral pruritus pathways and the involved pruritogens and will serve as the focus of this review. Future studies may be required to evaluate the central processing pathways of pruritus specific in PDs.

### 4.1 Histamine in pemphigoid diseases

Histamine, one of the most well‐characterized pruritogens, is released mainly by mast cells, but it also comes from epidermal keratinocytes, basophils, and neutrophils.^24^, ^25^, ^26^, ^27^ Released histamine binds to H1 and H4 receptors on the cutaneous sensory neurons, resulting in the opening of transient receptor potential vanilloid 1 (TRPV1) channels that induce neuronal calcium influx to trigger acute pruritus.^28^ Antihistamines selectively targeting H1 receptors are frequently used to treat pruritus in dermatologic disorders, but the efficacy depends on the specific disease: i.e., effective in urticaria; but with limited efficacy in atopic dermatitis.^29^ While functional analysis for the H4 receptor has been conducted less than the H1 receptors, its contributions to pruritus have been elucidated in recent animal and clinical studies.^30^ For example, the blockade of H4 receptors displayed antipruritic and anti‐inflammatory effects in atopic dermatitis,^31^, ^32^ possibly through the inhibition of both direct sensory nerve stimulation and increased IL‐31 and thymic stromal lymphopoetin (TSLP) production by inflammatory cells.^33^, ^34^, ^35^

**FIGURE 1:**
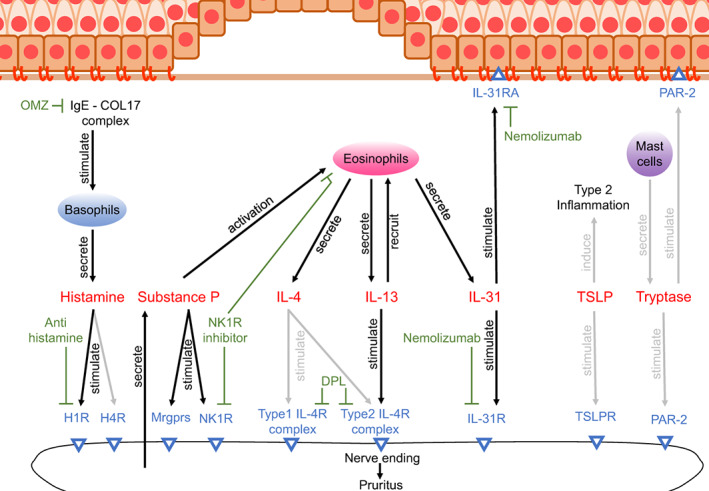
Multiple pruritogens in pemphigoid diseases. Histamine induces pruritus through H1 and H4 receptor (H1R and H4R) activation on the cutaneous sensory neurons. Basophils secrete histamine under the stimulation of IgE‐COL17 complex. Omalizumab (OMZ) and antihistamine target free IgE and H1R, respectively. Substance P induces pruritus directly through activation of the mas‐related G protein‐coupled receptors (Mrgprs) and neurokinin 1 receptor (NK1R) on the sensory neurons and indirectly through NK1R activation on eosinophils. NK1R inhibitor blocks NK1R on both neurons and eosinophils. IL‐4 and ‐13 induce pruritus through activation of type 1 and type 2 IL‐4 receptor (IL‐4R) complexes. Multiple types of the cells including eosinophils secrete IL‐4 and ‐13. IL‐13 recruits eosinophils. Dupilumab (DPL) blocks type 1 and type 2 IL‐4R complexes. IL‐31 stimulates IL‐31 receptor (IL‐31R) on neuron and epidermal keratinocytes. Multiple inflammatory cells including eosinophils secrete IL‐31 and nemolizumab blocks IL‐31R. Thymic stromal lymphopoetin (TSLP) may induces pruritus through both TSLP receptor (TSLPR) activation on the neuron and contribution to the release of type 2 cytokines. Tryptase secreted from mast cells may induce pruritus through activation of protease activated receptor‐2 (PAR‐2) on the neuron and epidermal keratinocytes. Gray arrows indicate pathways undefined yet in pemphigoid diseases.

In BP, high levels of histamine have been detected in blister fluids (Table 1).^36^ Several reports have indicated that mast cells, one of the major cellular sources of histamine, are infiltrated and degranulated in early lesions of BP.^37^, ^38^ However, others reported that the number of infiltrated mast cells in BP lesional skin is not increased compared to healthy controls and does not correlate with pruritus severity.^39^ Taken together, mast cell‐derived histamine likely does not play a significant role in BP‐associated pruritus or may contribute exclusively to the pruritus at very early stages. Basophils, which upon activation also releases histamine, are more infiltrated in BP lesions compared to healthy controls and correlate with itch severity (Table 1).^39^ As basophils in BP patients secrete histamine under the stimulation of IgE‐COL17 complexes through FcεRI,^40^ histamine from basophils may be a more important contributor to pruritus in BP than that from mast cells, especially in patients that have anti‐COL17 IgE (Figure 1).

**TABLE 1:** Summary of select pruritogens in pemphigoid diseases

Pruritogen	Mechanism/s	Findings in pemphigoid diseases	Treatments with promising efficacy or evidence	Treatments with limited efficacy/evidence or potential treatments
Histamine	Activation of H1/H4 receptors on cutaneous sensory neurons^28^	Increased levels in blister fluid^36^ Mast cells degranulated in early BP^b^, ^39^ Basophils more infiltrated in BP^a^, ^39^	Omalizumab (BP)^39^, ^42^, ^43^	Antihistamines^44^
IL4/−13	Induction of Th2 polarization and direct stimulation of cutaneous sensory neurons^52^, ^53^, ^54^, ^55^, ^56^, ^57^, ^58^	Increased levels in serum and skin in BP and MMPh^62^, ^63^, ^64^, ^65^ IL‐13 positive cells^a^ leading to eosinophil recruitment in BP^39^ Increased levels in serum (EBA passive transfer mouse model but not in human sera)^67^	Dupilumab (BP)^68^, ^69^, ^70^	Tralokinumab, lebrikizumab^72^
IL‐31	Activation of IL‐31 receptor A (IL‐31RA) and oncostatin M receptor (OSMR) on cutaneous sensory neurons^77^, ^78^, ^79^, ^80^, ^81^, ^82^, ^83^	Inconsistent serum levels in BP^74^, ^84^, ^85^ Increased levels in BP lesions and blister^b^ fluid^39^, ^74^ Increased protein levels of IL‐31 receptors (IL‐31RA and OSMR) in BP^a^, ^39^	–	Nemolizumab
Substance P	Activation of neurokinin 1 receptor (NKR1) and direct stimulation of cutaneous sensory nerves^89^, ^90^, ^91^, ^92^, ^96^, ^97^, ^98^, ^99^, ^100^	Increased Substance P‐positive cells^a^ and protein levels in BP^39^, ^107^ Increased NKR1+ cells in dermis^a^ in BP^39^	–	Aprepitant^101^, ^102^ Serlopitant^103^ Overpitant^105^ Tradipitant^106^
Tryptase	Activation of PAR‐2 receptor and direct stimulation of cutaneous sensory neurons^110^, ^111^, ^112^, ^113^	Elevated levels in BP blister fluid and sera^118^, ^119^, ^120^ Tryptase‐positive mast cells in BP^b^, ^39^ Increased tryptase‐positive cells in LAD^122^	–	Nafamostat
Thymic stromal lymphopoietin	Activation of TSLPR and IL‐7Rα and direct stimulation of cutaneous sensory neurons via TRPA1^127^, ^128^, ^129^, ^130^, ^131^, ^132^, ^133^	Increased in BP lesional skin, blister fluid, and sera^138^, ^139^	–	Tepelezumab
Endothelin‐1	Direct activation of cutaneous sensory neurons^148^, ^149^	Produces burning itch in humans, similar to BP^152^, ^153^, ^154^	–	Bosentan^152^, ^153^, ^154^

Abbreviations: BP, bullous pemphigoid; EBA, epidermolysis bullosa acquisita; H1/4, histamine 1/4 receptors; IL‐7Rα ‐ Interleukin‐7 receptor α; IL‐31, Interleukin 31; IL‐4/−13, Interleukins‐4/−13; LAD, linear IgA dermatosis; MMPh, mucous membrane pemphigoid; PAR‐2, protease‐associated receptor 2; PG, pemhigoid gestationis; Th2, T helper 2; TRPA1, transient receptor potential ankyrin −1; TSLPR, thymic stromal lymphopoietin receptor.

^a^ Positive correlation to pruritus severity,

^b^ No correlation to pruritus severity.

Clinically, while the effect of antihistamines is recognized to be limited, almost half of BP patients are given H1 receptor antagonists (Table 1).^5^ Detailed cohort studies are required to evaluate the effectiveness of antihistamines in PDs. Omalizumab (OMZ), a humanized IgG monoclonal antibody that binds to free human IgE, decreases histamine release from basophils (Figure 1).^41^ Several case reports and case series studies have documented OMZ treatment in BP, with about a half of the cases achieving complete disease control after several months on OMZ (Table 1).^42^, ^43^ Intriguingly, 64% of OMZ treated patients had relief of pruritus, suggesting that histamine release from basophils partially contributes to pruritus in BP and supports the aforementioned immunohistochemical analysis.^39^, ^43^

Antihistamines are routinely administered in clinical practice to other PDs such as LAD, PG, and EBA, however, its efficacy for these diseases has not been evaluated (Table 1).^44^ Successful treatment with OMZ in LAD with an immediate improvement of pruritus has been demonstrated in a few case reports.^45^, ^46^

### 4.2 IL‐4/−13 in pemphigoid diseases

Interleukins‐4 and ‐13 (IL‐4/−13) are crucial cytokines in type‐2 inflammation, mainly released from T helper type 2 (Th2) cells, eosinophils, mast cells, basophils, and natural killer cells.^47^, ^48^, ^49^, ^50^, ^51^ Both cytokines induce Th2 cell differentiation, M2 macrophage polarization, major histocompatibility complex (MHC) class II expression, B cell and plasma cell differentiation, and amplification of IgE production from plasma cells via the activation of their receptors.^52^, ^53^, ^54^, ^55^, ^56^ Moreover, both IL‐4/−13 directly stimulate cutaneous sensory neurons and lower their sensitivity threshold to other pruritogens through receptor‐mediated Janus kinase (JAK) activation.^57^, ^58^ Dupilumab (DPL) is a human IgG monoclonal antibody against the human IL‐4 receptor α chain (IL‐4Rα).^59^ Since IL4Rα constitutes type 1 (IL‐4 specific) and type2 (IL‐4/−13 specific) IL‐4R complexes, DPL inhibits IL‐4R signaling mediated by both IL‐4 and IL‐13. DPL is effective in treating pruritus in multiple diseases with Th2 polarization such as atopic dermatitis and prurigo nodularis.^60^, ^61^

In PDs, IL‐4/−13 are increased in both serum and lesions of BP and MMPh patients (Table 1).^62^, ^63^, ^64^, ^65^ The number of IL‐13 positive cells, but not IL‐4 positive cells, in the dermis correlated with the severity of pruritus in BP.^39^ IL‐13, rather than IL‐4, induces recruitment of eosinophils;^66^ therefore, IL‐13 is speculated to induce pruritus not only through direct stimulation of sensory nerve fibers but also through eosinophil‐mediated mechanisms (Figure 1). In EBA, serum IL‐4/−13 levels in EBA patients were undetectable; but an elevation of these cytokines was observed in the passive transfer mouse model of inflammatory EBA (Table 1).^67^ Since EBA encompasses a broad spectrum of clinical presentations (classical/mechanobullous form, inflammatory type (BP‐like EBA), mucous membrane‐EBA, IgA‐EBA, and Brunsting‐Perry type EBA);^22^ IL‐4/−13 levels in EBA patients may reflect the inflammatory characteristics that such subtype resembles; and may be elevated, for example, in BP‐like EBA.

Dupilumab has been utilized as treatment for BP based on some case reports and series (Figure 1 and Table 1). Interestingly, in several of these cases the pruritus improved faster, within a few weeks, compared to blister resolution which was typically observed after a few months.^68^, ^69^ More than 90% of the patients achieved a satisfactory response and more than a half of the cases achieved complete remission.^70^ A phase III clinical trial evaluating DPL in adult patients is ongoing (NCT04206553).^71^ For tralokinumab and lebrikizumab, monoclonal antibodies that target IL‐13,^72^ there are no published reports utilizing them as treatment of PDs as of the time of writing (Table 1).

### 4.3 IL‐31 in pemphigoid diseases

Interleukin‐31 is a member of the gp130/IL‐6 family cytokines and secreted from activated Th2 cells, eosinophils, mast cells, macrophages, and dendritic cells.^73^, ^74^, ^75^, ^76^ The cytokine acts as a ligand for the IL‐31 receptor A (IL‐31RA) and oncostatin M receptor (OSMR), which are expressed in multiple cells, such as T cells, DRG cells, keratinocytes, dendric cells, eosinophils, basophils, and macrophages.^77^, ^78^, ^79^, ^80^, ^81^, ^82^ Following the binding to the IL‐31‐receptor, the subsequent signaling controls chemokine release, proinflammatory cytokine secretion, and cell proliferation through JAK and mitogen‐activated protein kinase (MAPK) pathways. Additionally, IL‐31R is expressed on sensory neuron endings; thus IL‐31 also induces pruritus through direct stimulation.^83^ Nemolizumab, a humanized monoclonal antibody against human IL‐31RA, rapidly reduces pruritus in atopic dermatitis as early as day 2 with concomitant decrease in disease severity.^10^

In BP, while serum levels of IL‐31 show inconsistent results between currently published reports,^74^, ^84^, ^85^ increased IL‐31 levels were observed in infiltrating eosinophils of BP lesions and blister fluids (Table 1).^39^, ^74^ The number of the IL‐31 positive cells in the dermis did not correlate with pruritus severity;^39^ however, protein levels of IL‐31 receptors, IL‐31RA and OSMR, in both epidermis and dermis, were increased and correlated with pruritus severity. IL‐31R activation in both keratinocytes and inflammatory cells may induce pruritus through amplifying inflammation with proinflammatory cytokine release and production of pruritus mediators such as leukotriene B4 and thromboxane (Figure 1).^86^, ^87^, ^88^ There are no reports analyzing IL‐31 or IL‐31R in LAD or EBA.

Although there are no reports utilizing nemolizumab in BP or other PDs thus far, the aforementioned findings support its possible effectiveness in treating pruritus in BP (Figure 1 and Table 1).

### 4.4 Substance P in pemphigoid diseases

Substance P is a member of the tachykinin neuropeptide family and is secreted from nerve endings and inflammatory cells including eosinophils, neutrophils, basophils, T cells, macrophages, and dendritic cells.^89^, ^90^, ^91^, ^92^, ^93^, ^94^, ^95^ This neuropeptide binds to and activates the neurokinin 1 receptor (NK1R) and the mas‐related G protein‐coupled receptors (Mrgprs) on various cell types including nerve cells in the central and peripheral nervous system and multiple types of immune cells.^89^, ^96^, ^97^, ^98^ In inflammatory skin, substance P is released from both nerve endings and inflammatory cells, facilitating their interaction via autocrine and paracrine mechanisms. Substance P not only directly activates NK1R and Mrgprs on the nervous systems, but also stimulates the receptors on mast cells, basophils, and eosinophils, to release pruritogens such as IL‐4, IL‐13, and histamine.^92^, ^98^, ^99^, ^100^ Several NK1R antagonists have been developed and some of them were tested for their antipruritic activity.^101^ Aprepitant is a commercially available NK1R inhibitor approved for the treatment of vomiting and nausea associated with chemotherapy. Its antipruritic efficacy has not been consistently proven in clinical trials as the data remain conflicting.^101^ Topical application of aprepitant in chronic prurigo did not show significant improvement compared to the vehicle‐treated group.^102^ Serlopitant, another NK1R inhibitor, was originally developed for treatment of overactive bladder; however, the development for this indication has been terminated because of its inefficacy.^103^ As a potential treatment for pruritus associated with prurigo nodularis, atopic dermatitis, and psoriasis, serlopitant was initially tested in a large clinical trial (NCT03540160); however, the study was terminated by the company, citing that they are no longer pursuing its development for pruritus treatment.^104^ Other NK1R inhibitors, such as orvepitant and tradipitant, have been tested in clinical trials for the pruritus associated with epidermal growth factor receptor inhibitor and atopic dermatitis, respectively; however, neither of them demonstrated significant improvement of pruritus over placebo.^105^, ^106^

In BP, both protein levels in bister fluid and the number of infiltrating positive cells for substance P are increased (Figure 1 and Table 1).^39^, ^107^ Importantly, the number of the substance P‐positive cells in the dermis correlate with the severity of the pruritus.^39^ In addition, the number of NK1R‐positive cells in the dermis, mostly eosinophils, also correlates with the severity of the pruritus. Hence, NK1R inhibitors may be a potential effective treatment for pruritus in BP but there are no reports utilizing it for BP thus far (Table 1). Substance P or NK1R has not been evaluated in EBA or LAD.

### 4.5 Tryptase in pemphigoid diseases

Tryptase is a serine protease secreted from mast cells, which can activate protease‐activated receptor 2 (PAR‐2) in neuron and other cells.^108^, ^109^ Through PAR‐2 dependent and independent mechanisms, tryptase induces cytokine and chemokine release, extracellular matrix cleavage, mast cell activation, and neuronal calcium influx.^110^, ^111^, ^112^, ^113^ Tryptase induces pruritus via a PAR‐2 activated pathway, which was confirmed by inhibition of tryptase‐induced scratch behavior in mice using PAR‐2 antibodies or a PAR‐2 antagonist.^114^ Clinically, the link between pruritus severity and serum tryptase was established in mycosis fungoides.^115^ Several tryptase inhibitors were evaluated in early clinical trials for allergy, asthma, colitis, and psoriasis; but none of them were approved for clinical use.^116^ Nafamostat, a serine protease inhibitor and potential tryptase inhibitor used for anticoagulation and the treatment of pancreatitis, had efficacy in addressing pruritus in NC mice, which mimics atopic dermatitis.^117^ However, no reports have been published showing its efficacy as pruritus treatment in humans.

In PDs, elevated tryptase has been detected in both blister fluids and sera of BP patients (Table 1).^118^, ^119^, ^120^ However, the number of tryptase‐positive cells (mast cells) did not correlate with the severity of pruritus in BP.^39^ The function of tryptase in pruritus in PDs has not been directly tested in humans and other animals. On the other hand, Kit/Scf‐independent mast cell deficiency did not reduce the severity of blistering nor inflammation in the BP‐like EBA mouse model.^121^ Since scratch behavior contributes to the induction of blistering in this model,^7^ mast cells and its specific protease, tryptase, may not be important pruritogens in PDs, or maybe exclusively a contributor at very early stages, as mentioned in the histamine section (Figure 1). There are no reports utilizing nafamostat in BP (Table 1). Increased number of the tryptase‐positive cells were detected in LAD; however, its function is unclear thus far.^122^

### 4.6 Thymic stromal lymphopoietin in pemphigoid diseases

Thymic stromal lymphopoietin (TSLP) is a cytokine released from non‐hematopoietic cells such as keratinocytes, epithelial cells, fibroblasts, mast cells, macrophages, and dendritic cells.^123^, ^124^, ^125^, ^126^ This cytokine is a critical mediator of type 2 inflammation, affecting the maturation, survival, recruitment, and activation of dendritic cells, T cells, B cells, neutrophils, mast cells, eosinophils, basophils, and innate lymphoid cells.^127^, ^128^, ^129^, ^130^ In addition, TSLP contributes to the release of proinflammatory type 2 cytokines such as IL‐4, −5, −9, and − 13, through a heterodimeric receptor comprising TSLPR and the IL‐7 receptor α‐chain, resulting in the activation of the JAK/signal transducer and activation of transcription (STAT) pathway.^127^, ^131^, ^132^ Through this receptor binding on nerve endings, TSLP directly induces pruritus by activating transient receptor potential ankyrin 1 (TRPA1).^133^ Moreover, TSLP promotes pruritus indirectly with T cell and eosinophil‐dependent systemic type 2 immune response as mentioned earlier.^128^ Tezepelumab is a human IgG monoclonal antibody against TSLP, approved for treatment of asthma by the United States Food and Drug Administration and European Medicines Agency.^134^ Despite the accumulated data supporting the function of TSLP as a therapeutic target for pruritus, tezepelumab could not achieve statistically significant improvements in pruritus for atopic dermatitis in Phase 2A clinical trial (NCT02525094); and the company has abandoned its development for this indication.^135^, ^136^, ^137^

Increased TSLP in lesional skin, blister fluid, and serum of BP patients has been reported in several studies (Figure 1 and Table 1).^138^, ^139^ Intriguingly, the deletion of NC16a domain of COL17 induced TSLP release from keratinocytes in both in vitro and mouse models.^140^ Although this NC16a deletion in COL17 is not reported in BP, the dysfunction of COL17 caused by its internalization or proteolytic degradation may be involved in the elevation of TSLP in BP. On the other hand, an immunohistochemical study has demonstrated that TSLP levels in the lesional epidermis do not correlate with pruritus severity.^39^ There are no reports of tezepelumab use in BP; and there are no studies examining TSLP levels in EBA, LAD, or other PDs.

### 4.7 Other possible pruritogens in pemphigoid diseases

Aside from the pruritogens mentioned above, multiple molecules have been found to cause pruritus in skin diseases; however, these molecules have not been analyzed well in PDs. On the other hand, there are several molecules identified in PDs that were suggested to trigger pruritus; additional detailed functional analysis is still required to confirm whether these molecules exhibit a true pruritic function. Recognizing such limitations, the characteristics of these molecules and their possible pathological functions in pruritus for PDs are enumerated and discussed below as avenues for further investigation.

In PDs, multiple proteases have been identified as contributors of blister formation by cleaving DEJ anchoring proteins.^11^ Some of these proteases such as neutrophil elastase, cathepsin S, chymase, and granzyme B can activate protease activated receptor‐2 (PAR‐2) in the same manner as tryptase.^141^, ^142^, ^143^, ^144^ Since PAR‐2 activation in sensory neuron evokes pruritus, these proteases may potentially contribute to pruritus in PDs. No direct evidence for pruritus induction has been identified for these proteases yet.

Endothelin‐1 is a potent vasoconstrictor peptide secreted from multiple cell types, including endothelial cells, dendritic cells, monocytes, macrophages, nerve cells, and keratinocytes.^145^, ^146^, ^147^ Endothelin‐1 also induces pruritus with direct activation of sensory neurons.^148^, ^149^ Coincidently, ET‐1 injection elicits burning itch in humans similar to the one reported in BP patients.^150^ Bosentan, an endothelin‐1 receptor antagonist, is used in the treatment of pulmonary hypertension.^151^ A recent animal study showed that the topical application of bosentan reduced scratch behavior in a mite‐induced dermatitis mouse model, suggesting that bosentan may decrease pruritus severity.^152^, ^153^, ^154^ The concentration of endothelin‐1 in PDs may not have been analyzed yet.

IL‐33 is a member of the IL‐1 family and a potent amplifier of type 2 immune responses, which is expressed in various cell types including endothelial cells, epithelial cells, and fibroblast‐like cells.^155^ This cytokine was recently proposed as an important pruritogen in itch in dry skin.^156^ On the other hand, in BP, the IL‐33 protein levels were reported to be low to undetectable in both blister fluid and serum.^157^ IL‐33 may not contribute to the pruritus in PDs.

## 5 CONTRIBUTION OF PRURITUS IN BLISTER FORMATION

In PDs, the skin lesions usually localize at the areas with friction, such as trunk, groin, and extremities, suggesting that scratching plays a role in lesion development. It was also noted that in the EBA mouse model, as the mice started to scratch and bite themselves at ears, head, face, neck, and leg, obvious blistering and eruption developed at the scratched areas.^158^ Recently, several mechanisms on how scratching induces skin lesions were suggested using these animal models. Using the EBA mouse model developed by passive‐transfer of fluorescent‐labeled anti‐mouse Col7 IgG, Hundt et al. revealed that mechanical irritation induced additional accumulation of pathological IgG at DEJ, followed by neutrophil recruitment at the skin lesions.^159^ As another mechanism, Niebuhr et al. demonstrated that epidermal damage induced Th1 polarization, triggering the inflammation at the skin further through the recruitment of autoreactive Th1 effector cells using an immunization‐induced EBA mouse model.^6^ A BP mouse model targeting BP230 established by splenocyte transplantation has demonstrated the contribution of skin damage on BP development, as skin damage increased morbidity and severity of the skin lesions.^160^ This study suggests that trauma to the skin may expose BP230, an intracellular autoantigen, for the induction and development of inflammation. Based on these studies, scratching behaviors may contribute to inflammation and blister formation with (i) recruitment of pathological autoantibodies to the DEJ, (ii) Th1 polarization and recruitment of autoreactive Th1 effector cells, and (iii) exposure of intracellular autoantigens. Possibly through inhibition of the scratch behavior and its subsequent pathological events, local anesthetics decreased disease severity in the EBA mouse.^7^ Hence, scratch behavior contributes to further inflammation in PD pathology; therefore, effective treatments for pruritus in PDs may decrease severity of PD inflammation.

## 6 CONCLUSION

As described above, histamine, IL‐4, IL‐13, IL‐31, substance P, TSLP, and tryptase possibly contribute to peripheral itch mechanisms in BP. Awaiting the clinical trial results for DPL in BP, future properly designed clinical studies are necessary to determine whether the other available therapies targeting these potential pruritogens are truly effective for pruritus in PDs. In addition, central pruritus pathways relevant to PDs need to be identified to find out if treatments targeting central neural pathways such as gabapentinoids and cannabinoids may have potential therapeutic value.^161^, ^162^ On the other hand, further studies are required to specify pruritogens in other PDs, such as EBA, LAD, and PG.

Significant advancements in pruritus research, such as in atopic dermatitis, paved the development of multiple new treatment options that have had a tremendous positive impact on patient care. Despite the paucity of evidence thus far, as the contributions of each pruritogen are being more defined, safe and effective inhibitors are being developed, and newer pruritogens are being identified, a similar progress is anticipated in the development of novel and effective therapeutics for pruritus in PDs, intended to improve disease severity and patient QOL.

## FUNDING INFORMATION

This research was supported by funding from Grants‐in‐Aid for Scientific Research from the Ministry of Education, Culture, Sports, Science, and Technology of Japan 21K08305 (SH), Lydia O'Leary Memorial Pias Dermatological Foundation (SH), JSID's Fellowship SHISEIDO Research Grant (SH), Osaka Medical Research Foundation for Intractable Diseases Grant 27‐2‐41 (SH).

## CONFLICT OF INTEREST

None declared.
